# Development of a Procedure for the Determination of the Buckling Resistance of Steel Spherical Shells according to EC 1993-1-6

**DOI:** 10.3390/ma15010025

**Published:** 2021-12-21

**Authors:** Paweł Błażejewski

**Affiliations:** Institute of Civil Engineering, University of Zielona Góra, Licealna 9, 65-417 Zielona Gora, Poland; P.Blazejewski@ib.uz.zgora.pl

**Keywords:** finite element method (FEM), buckling, post-buckling, shell, spherical shells, design recommendations, buckling resistance, capacity curve

## Abstract

This paper presents the process of developing a new procedure for estimating the buckling capacity of spherical shells. This procedure is based entirely on the assumptions included in the standard mentioned, EN-1993-1-6 and also becomes a complement of EDR5th by unifying provisions included in them. This procedure is characterized by clarity and its algorithm is characterized by a low degree of complexity. While developing the procedure, no attempt was made to change the main postulates accompanying the dimensions of the spherical shells. The result is a simple engineering approach to the difficult problem of determining the buckling capacity of a spherical shell. In spite of the simple calculation algorithm for estimating the buckling capacity of spherical shells, the results obtained reflect extremely accurately the behavior of real spherical shells, regardless of their geometry and the material used to manufacture them.

## 1. Introduction

Shell structures have been the subject of academic research studies around the world for decades. Theoretical considerations on the buckling capacity of coatings were carried out already in the early 20th century [1,2] and in later years [3,4,5,6]. The development of research on spherical coatings increased in the second half of the 20th century. Between 1950 and 1980, a number of valuable studies were carried out, which resulted in a more accurate estimation of the buckling capacity of spherical coatings [7,8,9,10,11,12,13,14,15,16,17,18,19,20,21,22]. It was also during this time that computer analysis became increasingly important [23]. The use of computational methods for engineering calculations gave researchers the ability to study the behavior of spherical coatings to an extent not seen before [24,25,26,27,28,29]. Geometric imperfections began to be explicitly taken into account in coating research and advanced numerical analyses were used [30,31,32,33,34,35,36,37].

The use of the finite element method [38] in the study of steel spherical shells loaded by external pressure is crucial because of the ability to analyze the entire spatial system without having to make and study a physical model. Working on complete discrete models greatly speeds up the research and allows for the analysis of spherical shells of any geometry, material, support conditions, or loading. This kind of flexibility in research is very important because of the nature of work of spherical shells and the phenomenon of local or global buckling, which most often determines their load capacity. The loss of stability in question in this type of structure, in most cases, manifests itself locally and results in a rapid reduction in stiffness and, in extreme cases, leads to permanent deformations equivalent to structural failure. The form of buckling depends, among other things, on the above-mentioned conditions and geometric imperfections occurring in the analyzed shell [30,31,32,33,34,35,36,37]. The criterion of stability in many cases turns out to be the most important criterion of resistance capacity and at the same time the most frequent cause of failure. The phenomenon of loss of stability in spherical shells, and the geometric-material relations which determine it, are still the subject of research and scientific consideration [39,40,41,42,43], and one of these aspects is described in this paper.

## 2. The Subject of the Study

The lack of simple procedures for estimating the buckling capacity of steel spherical shells in the provisions of the current EN 1993-1-6 [44] and the use of GNA and GMNA numerical analyses, not recommended by the standard, in the creation of the calculation algorithm presented in EDR 5th Edition [45] has become a reason to conduct research related to the development of recommendations which are not burdened with such shortcomings. Two primary objectives were set:

First, to develop a procedure for estimating the buckling capacity of steel spherical shells subjected to external pressure: a procedure that complies with the provisions of Standard [44] EN 1993-1-6, which will be easy to apply, and will cover most spherical shells encountered in practice. The planned result of the work will be practical formulas allowing for an easy determination of the elastic buckling capacity *p*_Rcr_, the plastic capacity of the spherical shell *p*_Rpl_ and for an unambiguous determination of the other buckling parameters, i.e., *α*, *β*, *η*, *λ*_0_, needed to estimate the final buckling capacity of the shell. These are necessary components in the course of determining the load capacity, and their easy interpretation, reading and application will eliminate possible design errors. The new calculation procedure will be based on the same assumptions as the rest of the standard. Many forms of imperfections, which may adversely affect the buckling resistance of spherical shells, will be taken into account in the calculations.

Second, the unification of provisions contained in [45] European Design Recommendations 5th Edition within the scope of numerical analyses was used. At present, only the 15th chapter of this publication, which deals with the design of spherical shells, is based on numerical analyses of GMA and GMNA type, which is contrary to the idea of the standard [44] EN 1993-1-6.

All the analyses in the field of shell stability presented in this paper were carried out with the use of an advanced computer program COSMOS/M [38] in which calculations are performed on the basis of the finite element method (FEM). This application allows the performance of various numerical analyses, from static linear-elastic analyses to nonlinear buckling analyses.

In order to develop a complete procedure for estimating the buckling capacity of steel spherical shells, it is necessary to consider the full range of such structures most commonly encountered in engineering practice.

**Figure 1:**
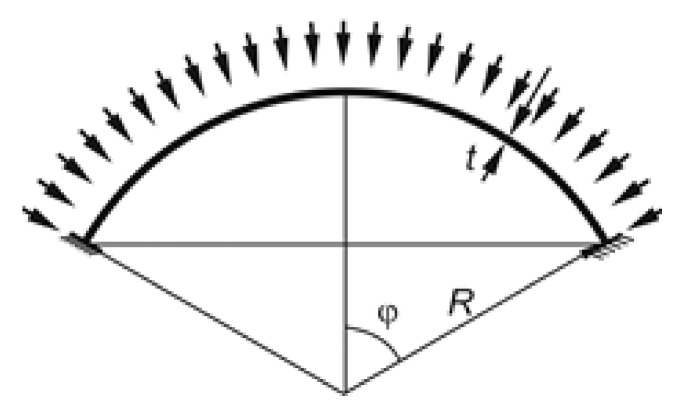
Geometric parameters of the spherical shell.

The shells were divided with respect to:

- shell opening angle *φ* = 10°, 20°, 30°, 45°, 60°, 90°—this is half of the angle determined by the extreme radii of the shell section (Figure 1),
- *BC* boundary conditions—one type of boundary conditions will be analyzed: *BC2*—rigid attachment
- varied value of the ratio of the shell radius *R* to its thickness *t*.

The following thicknesses *t* were considered in the analyses: 8.0 mm; 10.667 mm; 13.333 mm; 16.0 mm; 20.0 mm; 26.667 mm, which, given a constant shell radius *R* = 8000 mm, gives an *R/t* ratio of: 1000, 750, 600, 500, 400, 300, respectively.

First, LBA analyses (linear buckling analyses) were performed for the whole series of shells depending on the angle *φ*, boundary conditions (*BC)* and *R/t* ratio. The results of these analyses allowed the derivation of the formula for the elastic buckling capacity of spherical shells *p*_Rcr_.

The second stage is the determination of the plastic capacity of the shell *p*_Rpl_, which can be estimated on the basis of MNA (material nonlinear analysis) analyses performed for the whole series of shells.

In the third stage, a geometrically and materially nonlinear numerical analysis was performed, which took into account imperfections. It should be noted that which form of imperfection causes the most unfavorable situation was not known: i.e., results in the lowest value of buckling capacity. The following imperfection forms were analyzed

- imperfection in the form of a first and second form of loss of stability;
- local buckling imperfection—a buckling of two sizes to verify that the size of the local buckling has a significant effect on the buckling resistance;
- imperfection in the form of meridional spalling of the shell;
- flattening imperfection of the spherical shell top.

Individual geometric imperfections were considered in the range of amplitudes: 0.25*t*, 0.5*t*, 1.0*t*, 1.5*t*, 2.0*t*. The determination of the lowest value of the spherical shell resistance from the GMNIA analyses was a key step because of the need to estimate the remaining buckling parameters: *α*, *β*, *η*, *λ*_0_.

Collecting the set of buckling parameters values, obtained from the whole series of analyses depending on the angle *φ*, *R/t* ratio, boundary conditions (*BC*), imperfection type, and imperfection amplitude, allowed the creation of a uniform procedure for determining the buckling capacity of spherical shells.

## 3. Use of FEM Analysis

The use of advanced computer programs based on the finite element method (FEM) appears to be a key element in the process of testing spherical shells. The ability to perform global computer analyses speeds up the design process, but, most importantly, increases reliability and accuracy. The program itself, which was used to perform these analyses, is also not without significance. In the research conducted, the author, in the main, used the COSMOS/M system.

The finite element that was chosen for the analyses is a four-node quadrilateral surface element called SHELL4T. This element allows for both linear (LBA) and nonlinear (MNA, GNA, GMNA, and GMNIA) numerical analyses.

The material properties that have been assigned to the SHELL4T finite element correspond to steel. Their values, depending on the analysis that was performed, are as follows:

- Young’s modulus of elasticity *E* = 205 GPa,
- Poisson’s ratio *v* = 0.3,
- strengthening modulus *E*_T_ = 0 Pa,
- characteristic yield stress *f*_yk_ = 235 MPa.

**Figure 2:**
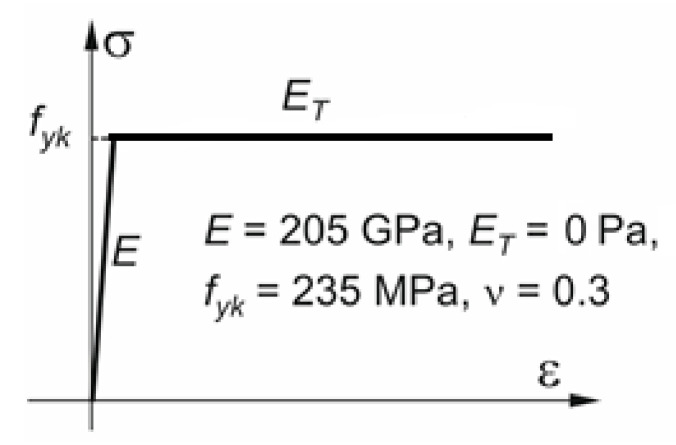
Material curve in MNA analyses.

In the LBA-type linear analyses, the material showed a linear relationship between stress and strain. The MNA analyses used a nonlinear characteristic of the material showing an *E*_T_ gain modulus of zero. The bilinear curve describing the material is shown in Figure 2.

Different shell thicknesses were assigned to the finite element according to the scope of the study. The thicknesses *t* were: 8.0 mm; 10.667 mm; 13.333 mm; 16.0 mm; 20.0 mm; 26.667 mm, which, with a constant shell radius *R* = 8000 mm, resulted in *R/t* ratios of: 1000, 750, 600, 500, 400, 300, respectively.

In each analysis, the spherical shell was loaded with an external pressure directed normally onto the surface. The set load value was 1.0 MPa. The application of the external load, unitary in value, resulted in the results in the form of load multipliers at which the analyzed shell plasticizes or loses its stability locally or globally. A schematic of the application of external pressure to the shell surface is shown in the figure below.

**Figure 3:**
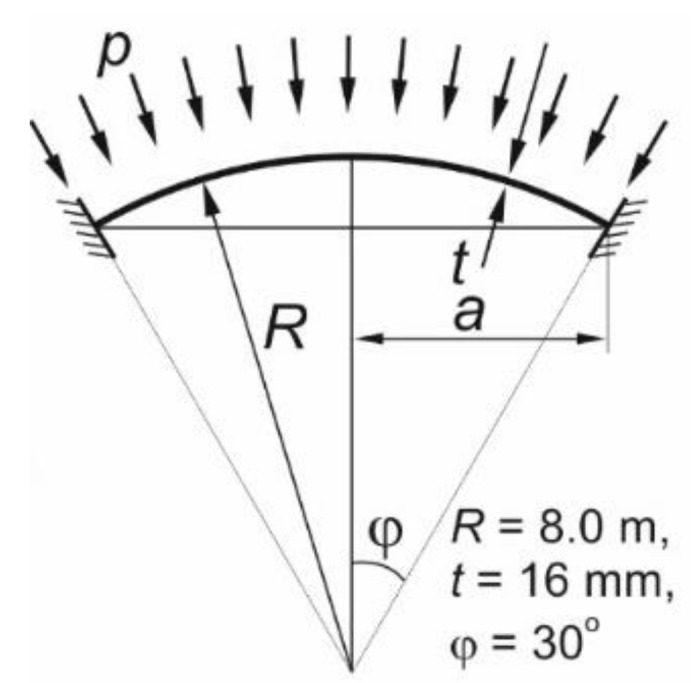
Shell loaded with external pressure. Supporting scheme.

The boundary conditions applied to the discrete models during the numerical analyses of the spherical shells corresponded to the most common support conditions encountered in engineering practice, namely: full restraint. This condition resulted in the locking of all 6 degrees of freedom at each node of the finite element mesh located at the edge of the shell. An idealized schematic of such a support is shown in Figure 3.

During the study of spherical shells, three types of numerical analyses were used. The first is linear elastic bifurcation analysis LBA, the second is physically nonlinear analysis MNA, and the third, and most advanced analysis is geometrically and physically nonlinear analysis with imperfections GMNIA. 

## 4. Linear Elastic Bifurcation Analysis LBA—Determination of Elastic Critical Load Capacity of *p*_Rcr_ Shell

Performing a linear elastic bifurcation analysis is necessary when considering spherical shells that are subjected to arbitrary loads and the critical buckling capacity is to be used in further analyses leading to an estimate of the ultimate buckling capacity. Existing standard provisions currently suggest the use of numerical analyses to determine the critical resistance *p*_Rcr_. This value is a reference parameter necessary for the calculation of the ultimate buckling resistance measure. The performance of LBA analyses, as well as the correct interpretation of the results obtained, is not always understood by the engineer performing the calculations. Therefore, it is advisable to develop an expression that unambiguously defines the critical buckling capacity, taking into account the geometrical parameters of the shell, i.e., shell thickness *t*, shell radius *R,* and half opening angle *φ*.

### 4.1. Description of Geometry and Boundary Conditions 

**Figure 4:**
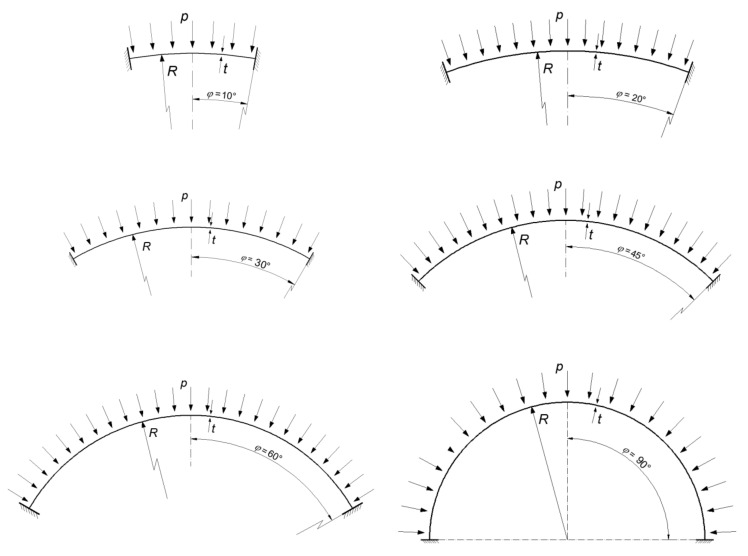
Geometrical diagrams of the analyzed spherical shells.

Spherical shells of varying geometries were analyzed, covering the range of shells most commonly encountered in engineering practice. It has been assumed that all the spherical shells under consideration are described by a constant radius *R* of *R* = 8000 mm, while the variable geometrical parameters are the half-angle of shell opening *φ* with the following values: 10°, 20°, 30°, 45°, 60°, 90°, and a shell thickness *t* of: 8.0 mm, 10.667 mm, 13.333 mm, 16.0 mm, 20.0 mm, 26.667 mm. The ratio of the shell radius to its thickness *R/t* gives us the values 1000, 750, 600, 500, 400, 300, respectively. The geometry diagrams together with the support conditions are shown in Figure 4.

The scope of the research carried out included one type of shell support: rigid support (restraint). The choice of this type of support is also reflected in engineering practice, as this type of restraint is the most commonly used solution by designers.

### 4.2. Results of Numerical Analysis of LBA 

Linear elastic bifurcation analyses LBA allow the determination of buckling forms of the system under consideration with their corresponding eigenvalues. They are based on the bending theory of thin-walled elastic shells of ideal geometry, taking into account small deflections and linear material characteristics. In this type of analysis, the initial geometry is free of any geometric imperfections and the material properties do not change during the analysis. The result of a correctly performed calculation is the pressure at which the shell ceases to be stable. This is the lowest value of the eigenproblem to which linear elastic stability analysis reduces. At the same time, this value acts as a critical resistance multiplier *p*_Rcr_, i.e., the reference value for the case under consideration. A load corresponding to *p*_Rcr_ applied to the shell may lead to buckling and a significant change in geometry.

**Figure 5:**
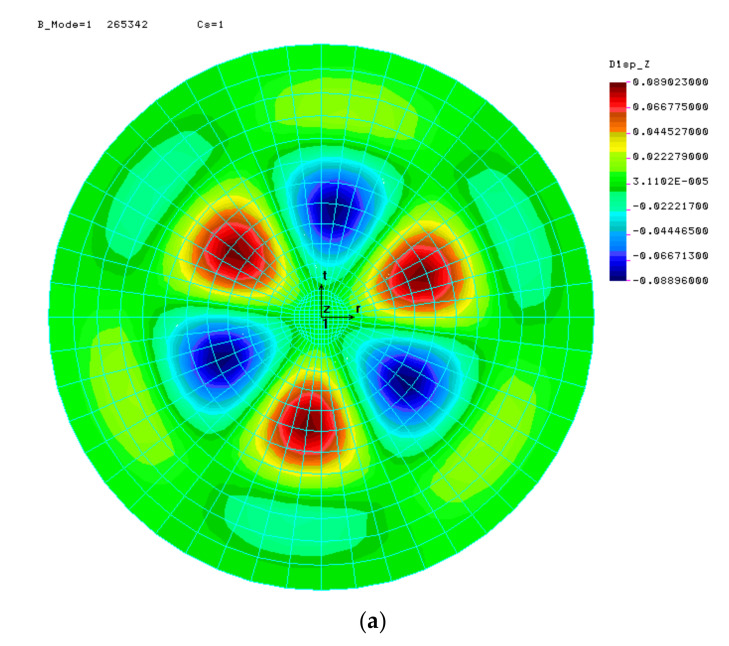

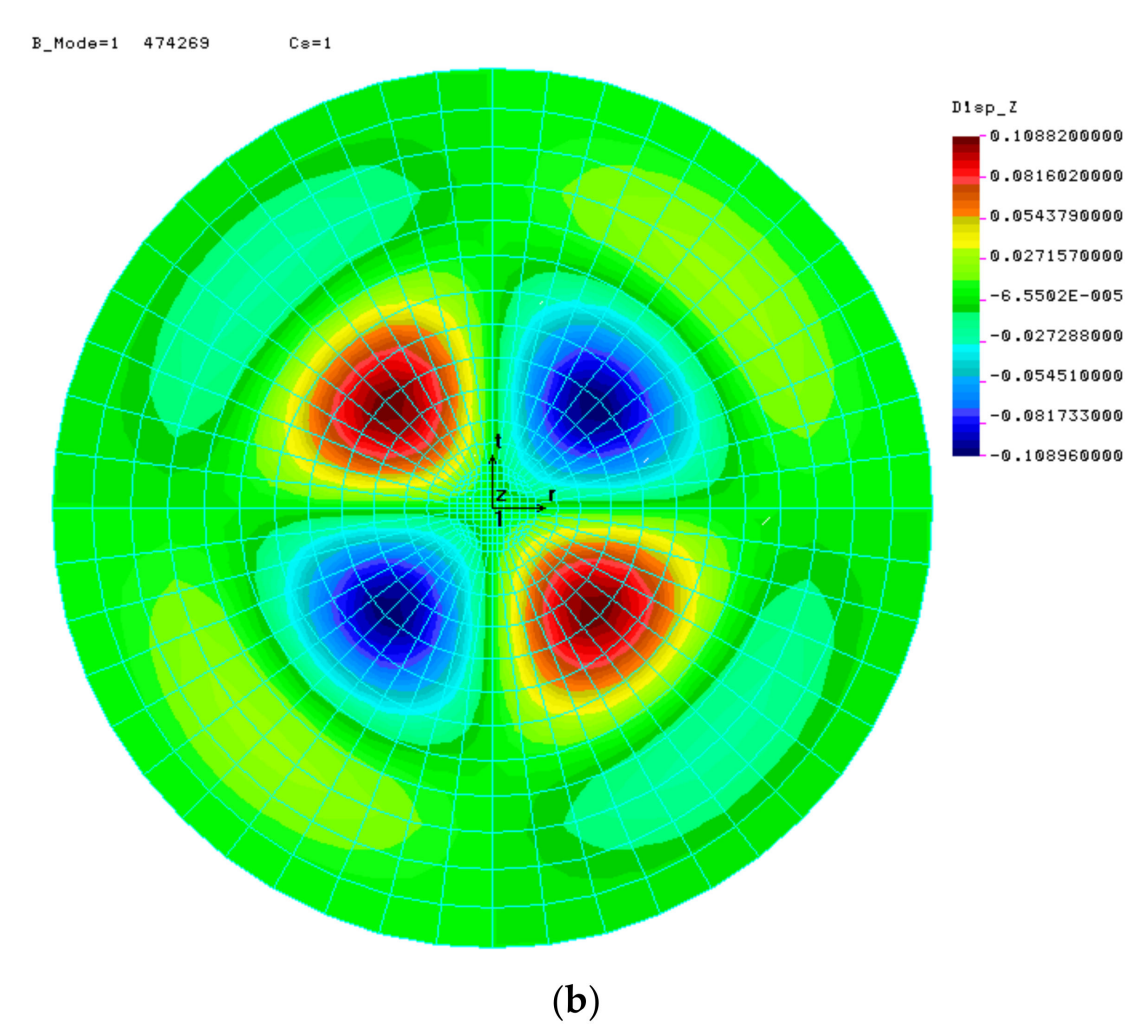
First form of loss of stability for shells with angle *φ* = 10° at (**a**) *t* = 8.0 mm, (**b**) *t* = 10.667 mm.

Several numerical analyses of LBA as a function of the half-angle of opening *φ* and the shell thickness *t* were carried out. In each case, axisymmetric results were expected and only these were considered correct. The results of calculations for the selected two cases are presented below, in the form of displacement maps *U*z, in Figure 5.

The results of all LBA analyses, for each of the angles analyzed, were collected and presented in tabular form. Table 1 illustrates the values of the critical pressure *p*_Rcr_, expressed in [MPa], as a function of the shell thickness t and the half-angle of opening *φ*.

### 4.3. Conclusions

**Figure 6:**
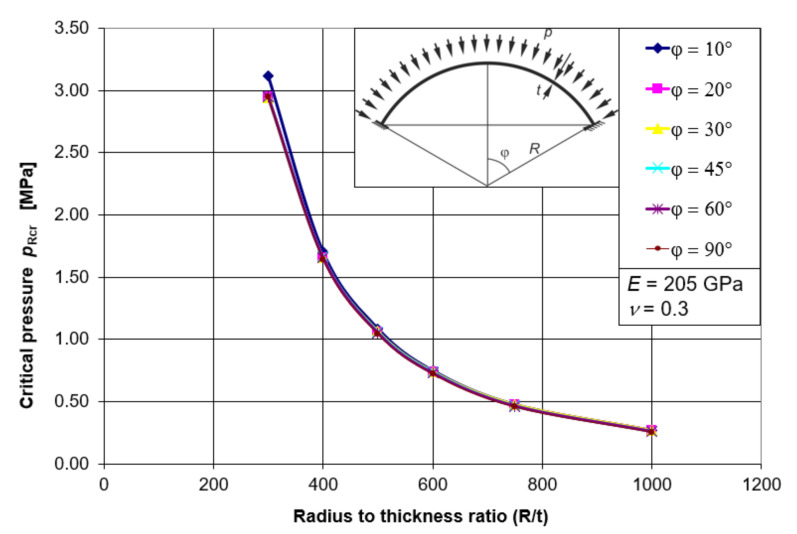
Critical pressure paths *p*_Rcr_ for all angles *φ*.

Putting all the results together, in the form of graphs (Figure 6), leads to an interesting observation. Namely, the value of the critical pressure of the spherical shell *p*_Rcr_ does not depend on the value of the half-angle of opening *φ*, and thus on the shell elevation. It depends only on the geometrical factor in the form of the ratio of the radius *R* and the shell thickness *t*, and on the material factor in the form of the assumed elastic modulus *E*. The observed relations enable the development of one simple mathematical formula describing the elastic buckling capacity of the shell *p*_Rcr_ as a function of *R/t*. This formula, within its scope of application, will include shells with a half opening angle in the range *φ* = 10–90°.

Using the best fit procedure in the Mathematica program, which takes into account the course of all *p*_Rcr_ (*R/t*) plots as a function of *φ*, a relatively simple formula was developed to describe the critical pressure *p*_Rcr_. This formula takes the form:

(1) $$p_{\mathrm{Rcr}}{}_{\left(\mathrm{LBA}\right)}=\;1.303E{\left(\frac{t}{R}\right)}^{2},$$

where *E* is to be substituted in [MPa].

The best fit procedure applied allowed the proposed formula to optimally fit all critical pressure paths, thus creating a path for the elastic buckling capacity of spherical shells rigidly fixed in the base.

The newly developed procedure for the estimation of the critical buckling capacity *p*_Rcr_ is based on numerical analyses recommended by EN 1993-1-6.

## 5. Physically Nonlinear Numerical Analysis of MNA—Determination of the Plastic Resistance of *p*_Rpl_ Shell 

Non-linear material analysis (MNA) allows for the determination of the plastic resistance of a *p*_Rpl_ shell. This resistance is used to estimate the plastic resistance when checking limit states LS1 and LS2. It is also used when checking the ultimate limit state due to buckling LS3, acting as a plastic multiplier of the resistance obtained by calculation as a plastic limit load. In these analyses, the material parameter *f*_yk_ is introduced, while bearing in mind that the effect of strain amplification must be neglected. The determination of the plastic resistance *p*_Rpl_ is essential for the estimation of the buckling capacity of the shell LS3, since, together with the critical resistance *p*_Rcr_, it serves to determine the generalized relative slenderness of the whole spherical shell.

### 5.1. Description of Geometry and Boundary Conditions 

The numerical analyses of MNA were based on the same spherical shell geometries used in the linear bifurcation analysis (LBA). As before, it was assumed that all the spherical shells under consideration are described by a constant radius *R* of *R* = 8000 mm, while the variable geometrical parameters are half-shell opening angles *φ* with the following values: 10°, 20°, 30°, 45°, 60°, 90°, and a shell thickness *t* of: 8.0 mm, 10.667 mm, 13.333 mm, 16.0 mm, 20.0 mm, 26.667 mm. The ratio of the shell radius to its thickness *R/t* is 1000, 750, 600, 500, 400, 300, respectively. The geometry diagrams along with the support conditions are shown in Figure 4.

### 5.2. Results of Numerical Analysis of MNA 

A physical (material) nonlinear analysis (MNA) aims to determine the plastic limit load. It is based on bi-linear elastic-plastic material characteristics. It uses the bending theory of shells with ideal geometry and small deflections. The highest load value obtained in the course of the calculations is treated as the reference plastic load capacity of the shell *p*_Rpl_. Correct and acceptable results were considered to be those in which axial symmetry was observed with respect to the deformed form of the shell tested, as well as a sudden increase in displacement on the equilibrium path for the analyzed node. This increase was due to the non-linear characteristics of the material exhibiting a zero-value *E*_T_ gain modulus.

The maximum along the equilibrium path reached for a displacement of 0.1 m is taken as a measure of plastic resistance. This means that the load capacity is exhausted and the plastic flow mechanism appears.

**Figure 7:**
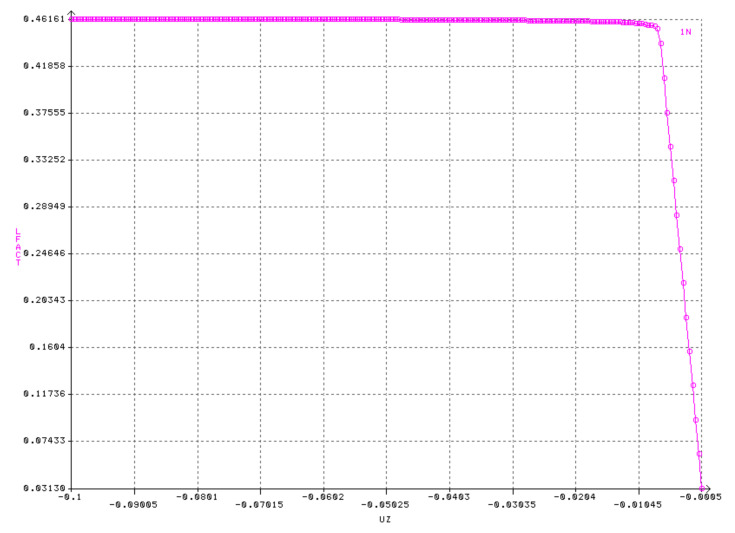
Equilibrium paths from MNA analyses for the node under consideration. Shell with angle *φ* = 30° and thickness *t* = 8.0 mm.

A number of numerical MNA analyses of shells with different half opening angles *φ* and with different shell thicknesses *t* have been carried out. These are shown below in Figure 7.

Selected results of calculations are presented (in the form of graphs showing equilibrium paths for the analyzed node). In most cases, node No. 1, located at the very top of the spherical shell, features in the results. The above results from the MNA analyses were collected and presented in tabular form. Table 2 presents the reference plastic resistance *p*_Rpl_, expressed in [MPa], as a function of the shell thickness t and the half-angle of opening *φ*.

### 5.3. Conclusions and Summary

**Figure 8:**
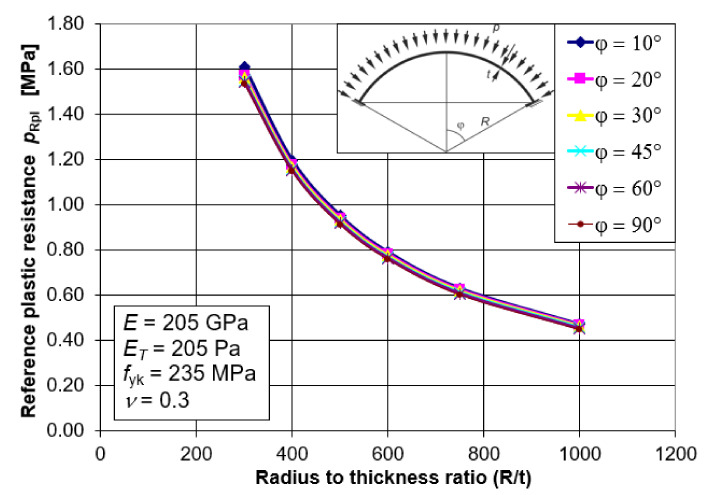
Values of the reference plastic resistance *p*_Rpl_ for all angles *φ*.

Examining all the graphs together (Figure 8), as was done for the LBA analyses, once again leads to an interesting observation. Namely, the values of the reference plastic resistance *p*_Rpl_ do not depend on the value of the half opening angle *φ*. They depend only on the geometrical factor in the form of the ratio of the radius *R* and the shell thickness *t*, and on the material factor in the form of the adopted yield strength *f*_yk_. The observed relations make it possible to develop one simple mathematical formula describing the reference plastic load capacity of the shell *p*_Rpl_ as a function of *R/t*. This formula, with its scope of application, will comprise shells with a half-angle between *φ =* 10°–90°.

Using the best fit procedure in Mathematica, which takes into account the course of all *p*_Rcr_ (*R/t*) diagrams as a function of *φ*, a relatively simple formula was developed to describe the reference plastic bearing capacity *p*_Rpl_. This formula takes the form:

(2) $$p_{\mathrm{Rpl}}{}_{\left(\mathrm{MNA}\right)}=\;1.986f_{\mathrm{yk}}\frac{t}{R}$$

where *f*_yk_ is to be substituted in [MPa].

The developed procedure for estimating the plastic resistance *p*_Rpl_, is based on numerical analyses recommended by PN-EN 1993-1-6. The development of an easy-to-apply formula that effectively describes the plastic resistance of a shell was the aim of the second part of the research and concludes the work on the reference plastic resistance of spherical shells.

## 6. Geometrically and Physically Nonlinear Numerical Analyses with GMNIA Imperfections 

Geometrically and physically nonlinear imperfection numerical analysis (GMNIA) is currently the most accurate method for determining the buckling capacity of the structure under analysis. This analysis is based on the theory of large deflections and the non-linear elastic-plastic characteristics of the material. It allows the determination of the elastic-plastic design capacity of the structure, taking into account the geometric imperfections of the system in an explicit manner. The deviations of the shape from ideal geometry, in the case of the spherical shells under consideration, may be caused by the manufacturing technology of these structures: in particular, irregularities near welded seams as well as the lack of ideal “sphericity” of the shell. In addition to the geometric imperfections resulting from the manufacturing practice, the imperfections corresponding to the first two buckling forms of the system analyzed are also taken into account. Each geometrical imperfection, regardless of its cause of origin, is considered with different values of amplitude. In these analyses, the ultimate level of exhaustion of the buckling capacity is considered to be the value of the load at which complete plasticization occurs. This value is considered to be the moment when the equilibrium path diagram for the node under consideration breaks clearly or takes the form of a horizontal line with no tendency to strengthen.

### 6.1. Types of Geometric Imperfections Considered 

The occurrence of any geometrical imperfection in a spherical shell leads to a reduction in the buckling resistance. The value by which the buckling resistance is reduced will depend on the form, size, and location of the imperfection. So far, numerical investigations on spherical shells have only considered imperfections in the form of the first, the second, or a combination of the first two buckling forms of the system. This approach is correct, however, it does not exhaust the issue of geometric imperfections. The fact of the occurrence of imperfections, due to the technology and precision of the construction of the shell, cannot be ignored and must be subjected to more extensive research.

In this article, the author presents the results of investigations carried out on spherical shells burdened with as many as seven different forms of geometrical imperfections. First, the imperfections resulting from the possibility of the appearance of imperfections connected with the quality and technology of the production of spherical shells are analyzed, and then the imperfections in the form of the first and second buckling form of the system analyzed are examined.

**Figure 9:**
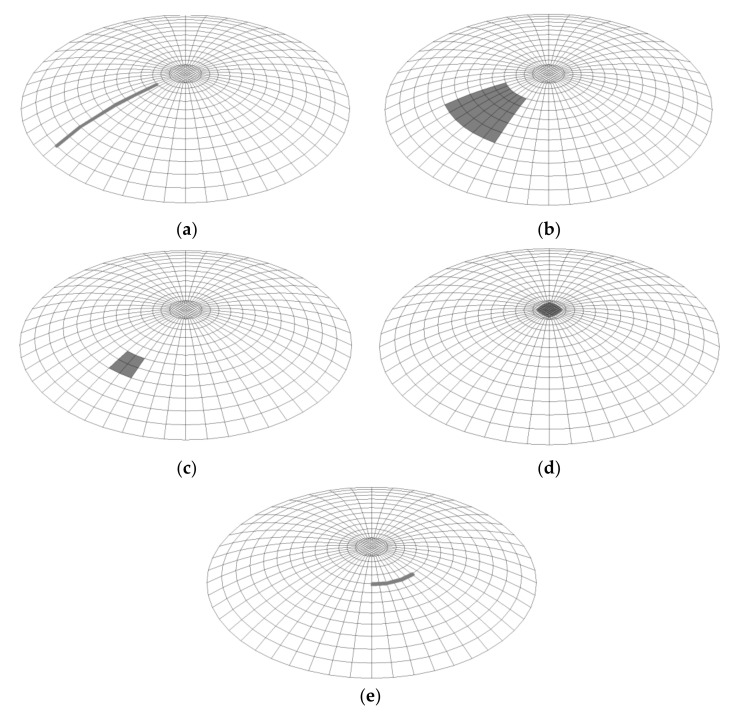
Location and form of imperfections: (**a**) meridional spline, (**b**) surface spline, (**c**) small surface spline, (**d**) surface spline on top of shell, (**e**) latitudinal spline.

The first geometrical imperfection investigated is the imperfection in the form of a linear meridional curvature, corresponding to possible imperfections created during welding works. This chamfer extends along almost the whole length of the shell formation (Figure 9a). The second imperfection corresponds to a surface casting of the spherical shell. This distortion is located in the middle third of the web length and has a surface area of approx. 2.10 m^2^ for a shell with an area of 65.63 m^2^. (Figure 9b). The third imperfection is also a surface spline, this time with a smaller area of influence, about 0.40 m^2^ (Figure 9c). The fourth imperfection is located at the top of the shell. It takes the form of a surface spline with an area of about 0.12 m^2^ (Figure 9d). The fifth imperfection corresponds to a linear latitudinal crimp and, like the first one, is supposed to simulate imperfections caused by welding work. It extends over a length of approximately one meter (Figure 9e).

**Figure 10:**
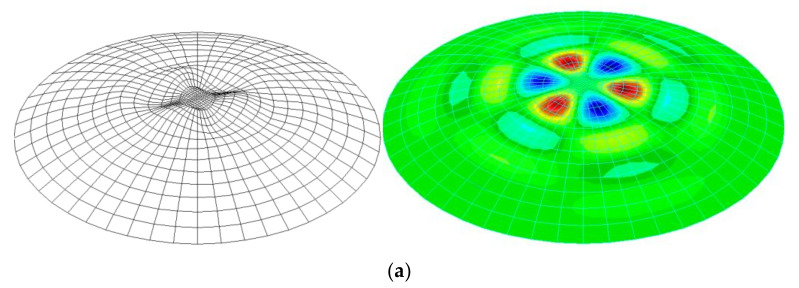

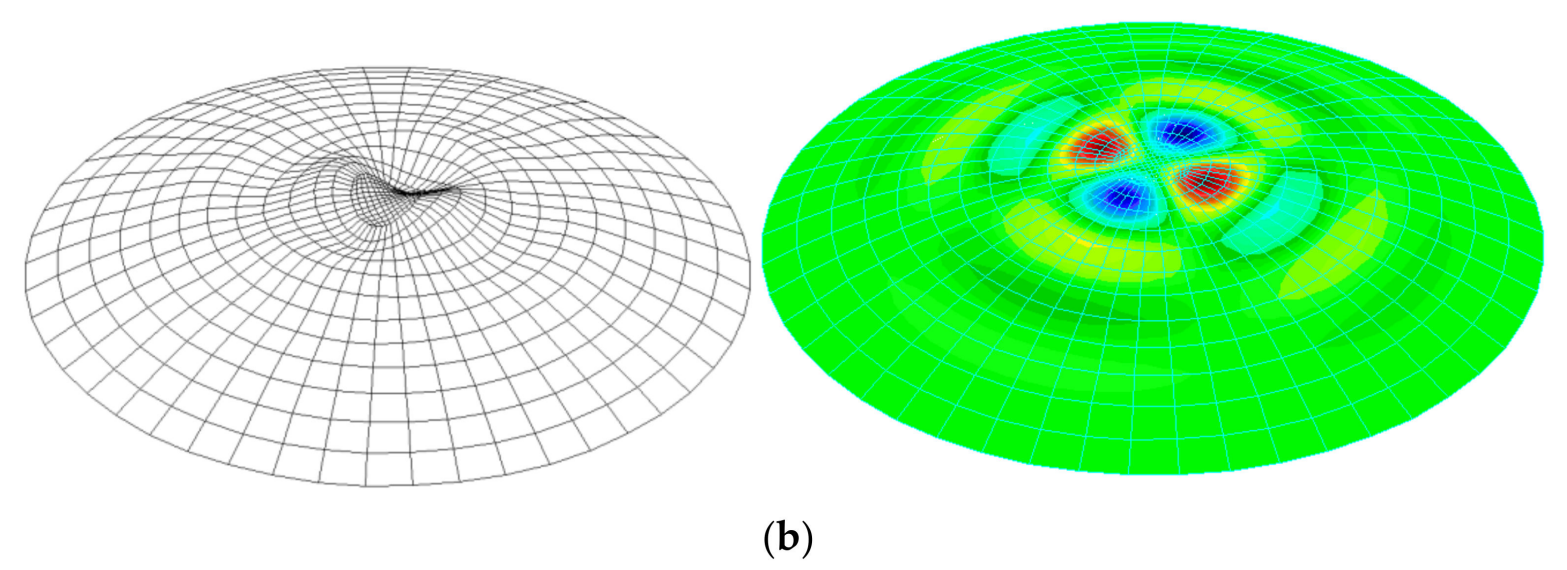
Geometric imperfection of the shell with angle *φ* = 30° and thickness *t* = 20 mm as: (**a**) first buckling form, (**b**) second buckling form.

The last two imperfections under consideration, No. 6 and 7, correspond to the first and second buckling forms of the analyzed shell, respectively (Figure 10a,b). These forms were obtained by analyzing a shell with wall thickness *t* = 20 mm and half opening angle *φ* = 30°.

### 6.2. Imperfection Amplitude—Performance Parameter Q 

The deformed forms corresponding to the first five forms of geometrical imperfections obtained by linear elastic static analysis and the two subsequent forms of imperfection obtained by linear bifurcation analysis are the basis for creating the initial geometries for the GMNIA analyses. The resulting deformed forms must be normalized to the desired imperfection amplitude. The magnitude of the imperfection amplitude is defined in the European recommendations for the design of shell structures (EDR 5th Edition) as:

(3) $$\Delta w_{k}=\;\frac{1}{Q}\sqrt{Rt}$$

where *R* and *t* are the shell radius and thickness, respectively. In turn, the manufacturing quality parameter *Q* depends on the manufacturing quality class adopted. Table 3 below shows the manufacturing quality classes and their corresponding *Q* parameters.

Bearing in mind that spherical shells of constant radius *R* equal to *R* = 8000 mm and variable thickness *t* equal to 8.0; 10.667; 13.333; 16; 20; 26.667 mm, respectively, each of the considered manufacturing quality classes will determine the necessity to normalize the magnitude of the amplitude of the considered geometrical imperfection depending on the size of *t* and the adopted parameter *Q*. The tables describing the above relationships and the value of the imperfection amplitude are presented below.

Imposing a specific form of imperfection on the initial geometry and scaling it to the desired amplitude was the starting point for performing geometrically and physically nonlinear numerical analyses with geometric imperfections, GMNIA for short. This condition was free of initial stresses, which is worth emphasizing. These analyses, as shown, depend on the half opening angle *φ*, the shell thickness *t*, the shell radius *R* (a constant value in the work), the imperfection form, the imperfection amplitude, and the manufacturing quality class adopted. Taking all of the above variables into account makes it necessary, at this stage, to perform around eight hundred GMNIA analyses. Therefore, it has not been possible to present all the individual results in this paper. Representative results of calculations for each manufacturing quality class will be shown and described.

### 6.3. Results of the GMNIA Numerical Analysis 

The creation of the geometry of individual imperfection forms with the desired amplitudes and the definition of a bilinear material was the basis for starting the GMNIA analyses. In these analyses, the ultimate level of exhaustion of the buckling capacity was considered to be the value of the load at which complete plasticization occurs. This value was determined when the graph representing the equilibrium path for the analyzed node (the node with the largest overall displacement within the imperfection) clearly broke or assumed the form of a horizontal line without any tendency to strengthen.

**Figure 11:**
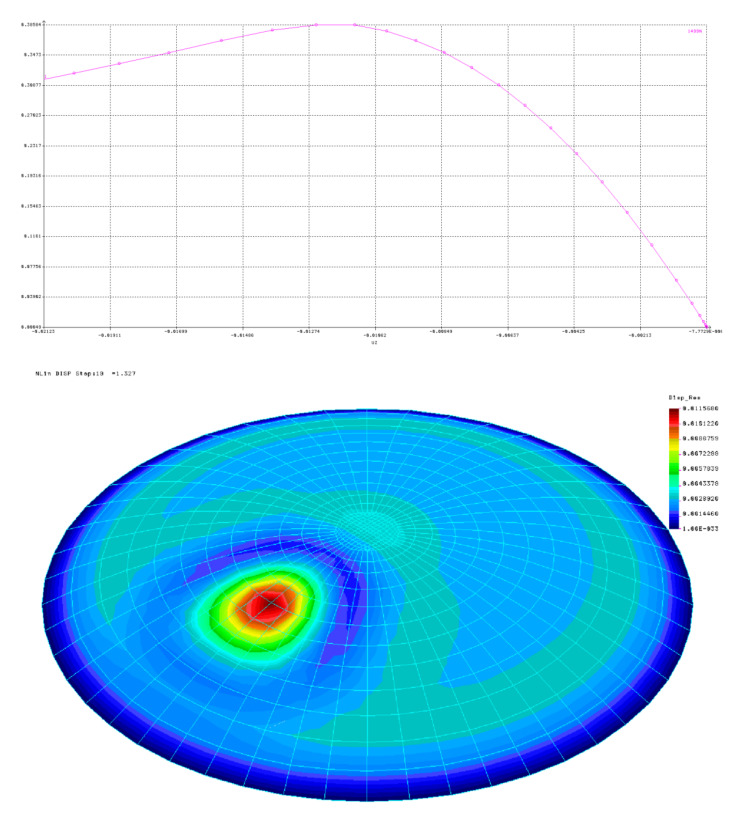
GMNIA analysis for shells with imperfection No. 2 with amplitude equal to 1.0*t*.

Figure 11 presents sample results of GMNIA analyses for the shell with half opening angle *φ =* 30°, shell thickness *t* = 16 mm, radius *R* = 8000 mm, yield stress *f*_yk_ = 235 MPa, modulus of elasticity *E* = 205 GPa, modulus of reinforcement *E*_T_ = 205 Pa and Poisson’s ratio *v* = 0.3. It is worth noting here that the adopted value of *E*_T_ is very small and served to eliminate singularities in numerical analyses, improving the stability of calculations. Figure 11 shows the equilibrium paths as well as the forms of deformation of the shell at the moment of complete exhaustion of the buckling capacity, as well as the values of generalized stresses resulting from the Huber–Mises–Hencky hypothesis. In this case, there is no doubt that the determination of the pressure level caused complete exhaustion of the buckling capacity of the analyzed system. In turn, the form of deformation at the final moment takes the expected shape, consistent with the declared imperfection.

#### 6.3.1. The Worst Geometric Imperfection 

Considering the results of GMNIA analyses with different imperfections of varying amplitude values, from 0.1*t* to 2.0*t*, one can observe a change in the worst imperfection form for the spherical shell investigated.

**Figure 12:**
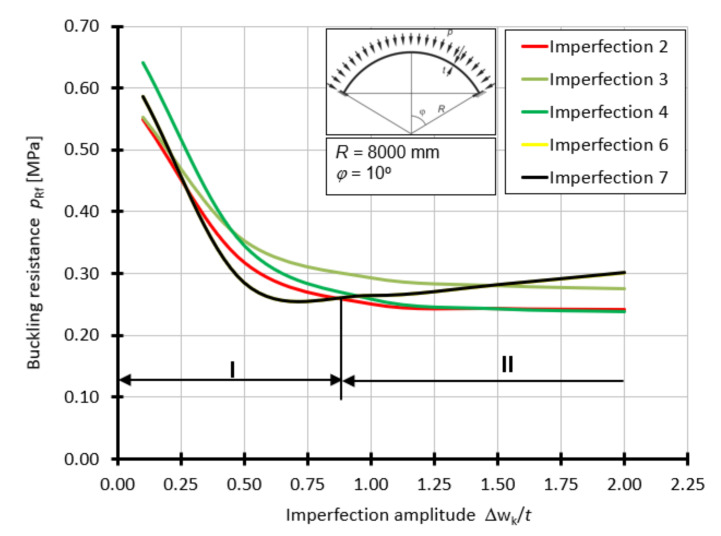
Buckling resistance *p*_Rf_ as a function of imperfection amplitude for shells with an angle *φ =* 10°.

For the first series of the considered shells with the half-angle of opening *φ =* 10°, the curves of the buckling capacity *p*_Rf_ as a function of *Δ*w_k_/*t*, depending on the form of geometric imperfection, are presented in Figure 12. For this case, the worst imperfections are imperfections 6 and 7 in the range of 0.1*t* to about 0.85*t*. In the range of 0.85*t* to 2.0*t*, the worst imperfection is in the form of a large spline (Figure 9b) or a spline located at the top of the shell (Figure 9d).

**Figure 13:**
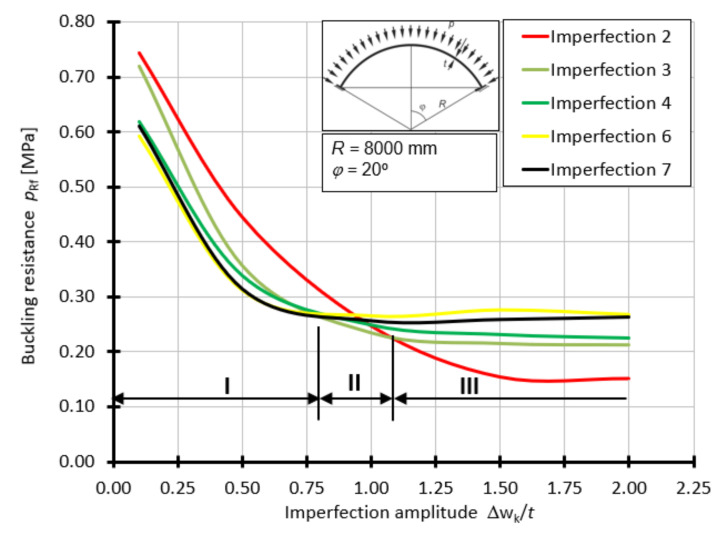
Buckling resistance *p*_Rf_ as a function of imperfection amplitude for shells with an angle *φ =* 20°.

For the shell with the half-angle of opening *φ =* 20°, the curves of buckling capacity *p*_Rf_ as a function of *Δ*w_k_/*t*, depending on the form of geometrical imperfection, are presented in Figure 13. For this case, the worst imperfections are imperfections 6 and 7 in the range of 0.1*t* to about 0.80*t*. In the range of 0.80*t* to 1.0*t*, the worst imperfection is in the form of a small lateral spline (Figure 9c), above the value of 1.0*t*, the worst imperfection is a large spline (Figure 9b).

**Figure 14:**
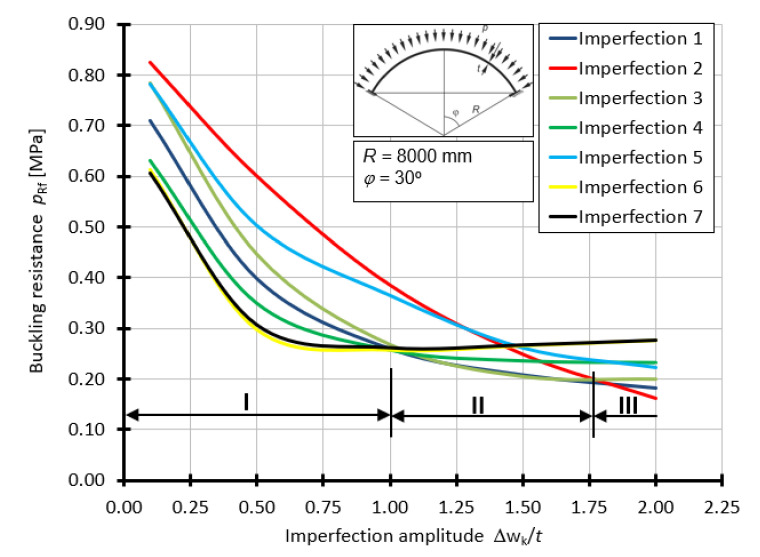
Buckling resistance *p*_Rf_ as a function of imperfection amplitude for shells with angle *φ =* 30°.

For a shell with a half-angle of opening *φ =* 30° (Figure 14), it is clear that in the range (I) from 0.1*t* to 1.0*t,* the imperfection that results in the lowest values of the buckling capacity of the shell is the imperfection in the form of the first and second buckling forms. Then, at a point with an amplitude equal to 1.0*t*, the levels of resistance *p*_Rf_ are equalized in most cases. The next clear interval (II) in which the worst imperfection becomes apparent is between 1.0*t* and 1.75*t*. In this range, imperfections 1 and 3 cause the greatest reduction in the ultimate buckling capacity. The occurrence of imperfections with an amplitude greater than 1.75*t* makes the worst form corresponding to a large superficial swelling. This is imperfection 2. In this range (III), a strong tendency towards a decrease in the value of the buckling load capacity of the shell can clearly be seen.

**Figure 15:**
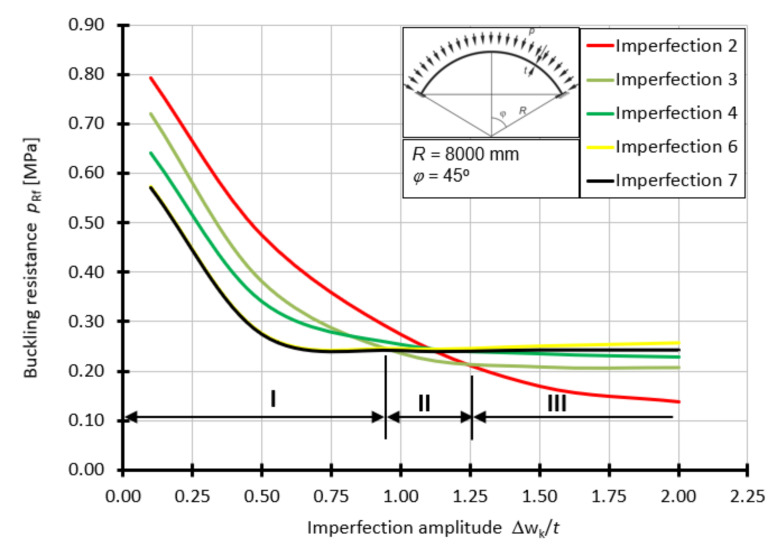
Buckling resistance *p*_Rf_ as a function of imperfection amplitude for shells with angle *φ =* 45°.

For the shell with the half-angle of opening *φ =* 45°, the curves of the buckling capacity *p*_Rf_ as a function of *Δ*w_k_/*t* depending on the form of geometrical imperfection are presented in Figure 15. For this case, the worst imperfections are imperfections 6 and 7 in the range of 0.1*t* to about 0.9*t*. On the other hand, in the range of 0.9*t* to 1.25*t*, the worst imperfection is in the form of a small lateral spline (Figure 9c), above the value of 1.25*t*, the worst imperfection is a large spline (Figure 9b).

**Figure 16:**
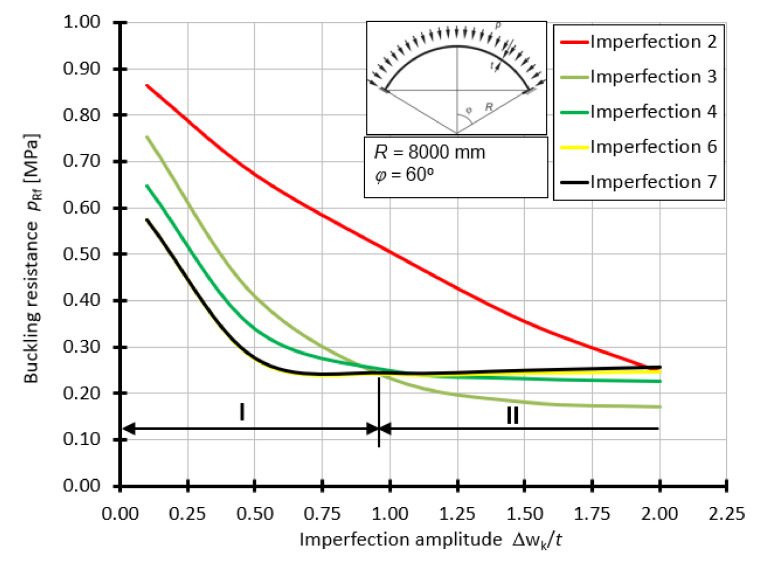
Buckling resistance *p*_Rf_ as a function of imperfection amplitude for shells with angle *φ =* 60°.

For the shell with the half-angle of opening *φ =* 60°, the curves of buckling capacity *p*_Rf_ as a function of *Δ*w_k_/*t*, depending on the form of geometrical imperfection, are presented in Figure 16. For this case, the worst imperfections are imperfections 6 and 7 in the range of 0.1*t* to about 0.9*t*. On the other hand, in the range of 0.9*t* to 2.0*t*, the worst imperfection is in the form of a small lateral curvature (Figure 9c).

The obtained results, in the form of graphs presenting the buckling resistance *p*_Rf_ as a function of the imperfection amplitude expressed by the *Δ*w_k_/*t* ratio, clearly show that imperfections in the form of the first and second buckling forms of the system cause the greatest, as far as value is concerned, reduction in the buckling resistance. This happens in the range of amplitudes 0.1*t* to 0.8 ÷ 1.0*t*, irrespective of the considered half-angle of opening *φ*, which is responsible for the shell loftiness. A further increase in the imperfection amplitude, to values greater than 1.0*t*, leads to the manifestation of other worse forms of geometric imperfections. These are imperfections in the form of a large surface spalling (over 2 m^2^), as well as spalling of a smaller area of influence (about 0.4 m^2^). The range in which they act as the worst geometrical imperfections is 0.8 ÷ 1.0*t* to 2.0*t*. Depending on the elevation of the spherical shell, one of them may dominate in the whole interval, e.g., imperfection 3 (Figure 16), or in certain parts of it, e.g., imperfections 2 and 3 (Figure 14).

#### 6.3.2. Modified Buckling Capacity Curve

An estimate of the ultimate buckling resistance of a spherical shell is possible when all the buckling parameters are known: *λ*_o_, *α*, *β*, *η*. The current EN 1993-1-6 does not give their values for spherical shells. This chapter is devoted to the dimensioning of spherical shells loaded with external pressure and contains an algorithm for their dimensioning by means of simple engineering formulae. In this procedure, the values of the buckling parameters are given: *λ*_o_ = 0.2; *β* = 0.7; *η* = 1.0. The parameter *α*, which is the reduction factor for elastic buckling, depends on the imperfection amplitude and is described by the formula:

(4) $$\alpha =\frac{0.7}{1+1.9\cdot {\left({\Delta w}_{k}/t\right)}^{0.75}}$$

where *Δ*w_k_ is the magnitude of the imperfection amplitude (Equation (3)); *t* is the shell thickness.

To achieve this, it is necessary to determine the above buckling parameters. This should be based on the numerical analyses recommended by the standard. This is the only way to obtain a consistent procedure for estimating the buckling capacity of spherical shells that meets the main requirements of the standard.

**Figure 17:**
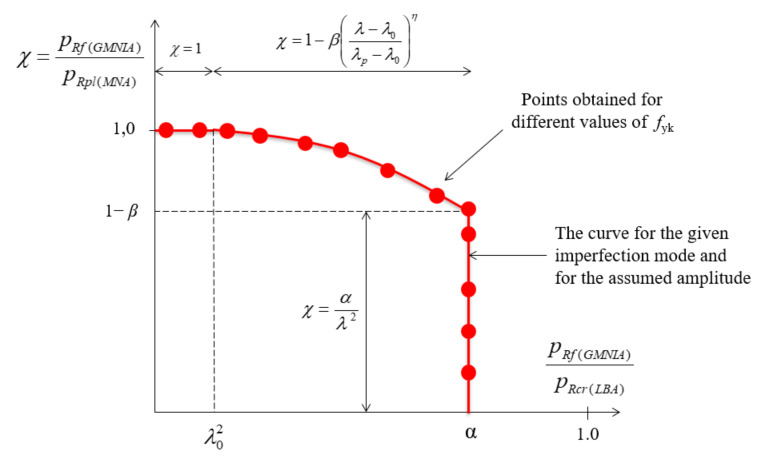
Idealized shape of the modified capacity curve.

The determination of modified buckling capacity curves, based directly on the conducted numerical analyses (LBA, MNA, GMNIA) enables the determination of the buckling parameters, which are necessary for the correct estimation of the buckling capacity with the use of calculation formulas. The method of its creation and obtaining of buckling parameters was described by Prof. J.M. Rotter in his publications [46,47]. In this procedure, we put on the abscissa axis the values resulting from the ratio of GMNIA/LBA analyses, and on the ordinate axis the values from GMNIA/MNA analyses. In this approach, the only variable is the characteristic yield stress value *f*_yk_. Assuming a different value of yield stress each time, we change the relative slenderness of the *λ* analyzed shell. The ideal shape of the modified load-carrying curve for a selected geometric imperfection of a specified amplitude is shown with a red line in Figure 17. Each successive red point corresponds to a different yield stress under consideration, and consequently to a different slenderness *λ*.

When carrying out the GMNIA and MNA analyses for very small values of yield stress *f*_yk_, one may expect the characteristic flattening of the graph (plateau), and the ordinate value determining the dimensionless reduction factor *χ* is 1.0. This is due to the fact that in such a shell, stresses equal to the yield stress leading to full plasticization will be reached faster than the critical buckling stress-causing elastic or elastic-plastic loss of stability. The shell is then in the plastic range. Increasing the yield strength in subsequent steps, we move to the elastic-plastic range of the shell operation.

This range is characterized by a parabolic course of the modified buckling capacity curve. The transition point between the two ranges is determined by the parameter *λ*_o_^2^ and corresponds to the moment of downward bending of the flat section of the modified curve. A subsequent increase of yield strength leads to a situation where the curve starts to take the form of a line pointing vertically downwards. This is because an increase in yield strength is no longer followed by an increase in ultimate buckling capacity obtained from the GMNIA analysis. The location on the axis of ordinates of the transition point to a vertical downward line determines the value of 1−*β*, while the projection of the vertical line on the axis of abscissa determines the parameter *α*. In this case, we are dealing with the elastic range of the shell.

**Figure 18:**
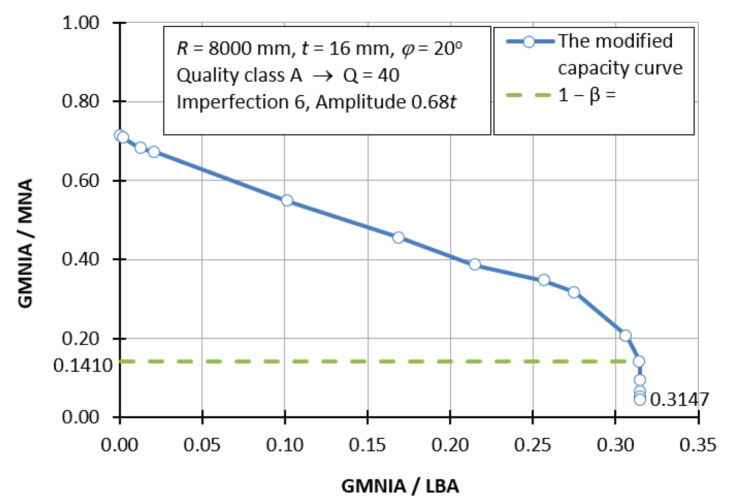
Modified buckling capacity curve for a shell with manufacturing quality class A, Q = 40, *φ* = 20°, *R* = 8000 mm, *t* = 16 mm, imperfection 6, amplitude 0.68*t*.

Figure 18 shows one example of a modified load curve. Such a limitation is dictated by the fact that such modified load curves are created for each individual case and depend on many parameters, i.e., manufacturing quality class (A, B, C), shell half-angle *φ*, shell thickness *t*, imperfection form, and imperfection amplitude. Each of the following diagrams corresponds to a different manufacturing quality class, the shells have different angles *φ* and are subject to a different form of imperfection with different amplitudes.

The reading of the buckling parameters *α* and *β* of the modified buckling curves created (and there were about 530 of them) was the characteristic flattening of the graph observed. This may suggest that none of the analyzed shells entered the ductile range of work, even when defining a material with a very low yield stress value. Therefore, there is no buckling parameter *λ*_o_^2^ indicating the transition point between the plastic and elastic-plastic range.

#### 6.3.3. Classical Capacity Curve

The classical capacity curve describes the behavior of a shell in three ranges of its work. In the plastic, elastic-plastic and elastic range. It also defines the relationship between the dimensionless reduction factor and the relative slenderness of the shell *λ*. To create it, it is necessary to estimate the relative slenderness for the particular case under consideration. This slenderness is obtained from the formula:

(5) $$\lambda =\sqrt{\frac{p_{\mathrm{Rpl}}}{p_{\mathrm{Rcr}}}}\;$$

where *p*_Rcr_ is the critical load capacity of the shell obtained from linear bifurcation analysis (LBA), taking into account the linear nature of the material and the ideal geometry of the shell. *p*_Rpl_ is the plastic load capacity obtained from physical nonlinear numerical analysis (MNA). Both values can be considered as reference values in the procedure for estimating the buckling capacity *p*_Rk_. Both procedures conform to the assumptions of EN 1993-1-6.

The buckling reduction ratio *χ*, which is the ratio between the characteristic buckling resistance *p*_Rk_ (GMNIA analysis) and the plastic resistance *p*_Rpl_ (MNA analysis), can be used as a dimensionless resistance to be plotted on the axis of ordinates when plotting a classic resistance curve.

**Figure 19:**
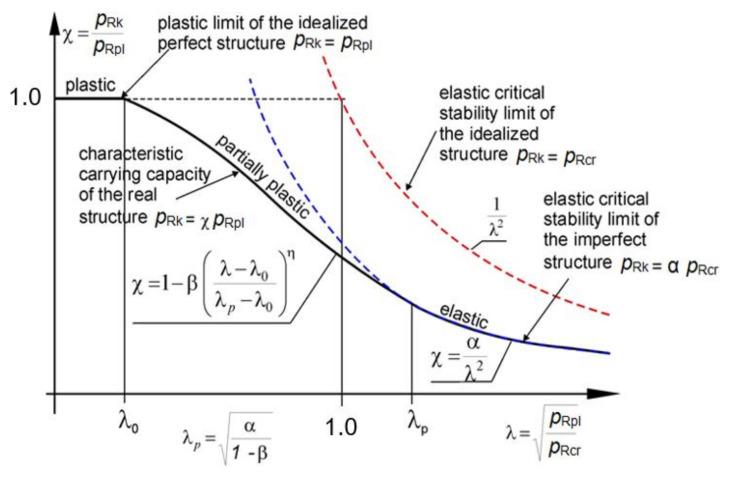
Ideal shape of classic buckling capacity curve.

The shape of the classic capacity curve is shown in Figure 19. On this curve, the relative slenderness of the shell *λ* can take values from three work ranges of the shell.

In the first of them, when *λ* < *λ*_0_ dimensionless bearing capacity *χ* = 1.0 we are dealing with a purely plastic range of work of the shell. This means that the destruction of the shell will occur only through plasticization of the cross-section. A characteristic flattening manifests itself on the graph. This occurs when the characteristic buckling resistance *p*_Rk_ is equal to the plastic resistance *p*_Rpl_. The end of this flattening is determined by the buckling parameter *λ*_0_.

The second interval, in the range of relative slendernesses *λ*_0_ < *λ* < *λ*_p_ determines the elastic-plastic behavior of the shell. Then, the dimensionless load capacity, according to PN-EN, is determined by the formula:

(6) $$\chi \;=\;1\;-\beta {\left(\frac{\lambda -\lambda_{0}}{\lambda_{p}-\lambda_{0}}\right)}^{\eta }$$

This interval is limited by the limiting relative slenderness *λ*_p_, which defines the transition point between the elastic-plastic and the elastic range. It can be determined from the compatibility condition of the function *χ* (*λ*) in the second and third intervals. It is equal to:

(7) $$\lambda_{p}=\sqrt{\frac{\alpha }{1-\beta }}\;$$

where the parameters *α* and *β* are known and have been determined using the modified resistance curve. The next value needed to be estimated is the exponent *η*, which can be determined directly from the formula:

(8) $$\eta \;=\;\frac{\ln(1-k)-\ln\beta }{\ln(\sqrt{\alpha }-\lambda_{0})-\ln\left(\sqrt{\frac{\alpha }{1-\beta }}-\lambda_{0}\right)}\;$$

**Figure 20:**
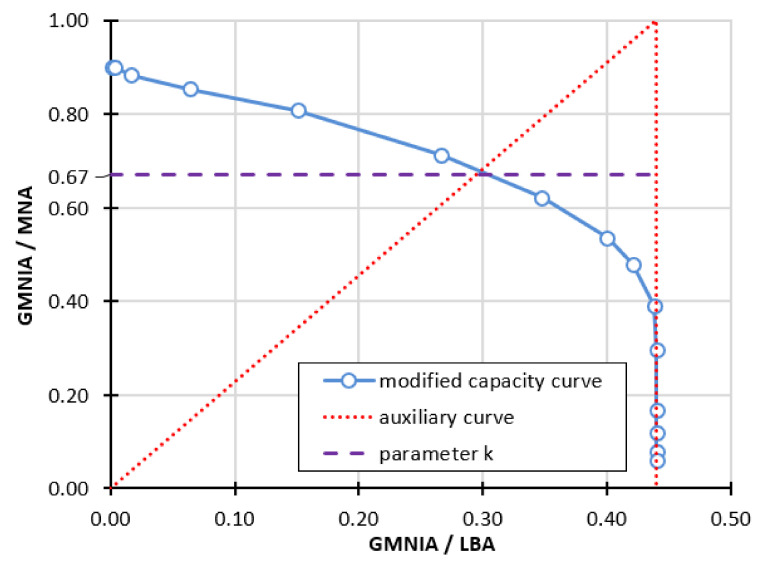
Method of determining the auxiliary parameter *k*.

In this formula, it is necessary to specify the auxiliary parameter *k*, which is defined as the point of intersection (Figure 20—dashed violet line) of the modified load-carrying curve with the straight segment connecting the point (0,0) and (*α*,1), shown by the red dotted line (Figure 20).

The third range of operation corresponds to the interval of relative slenderness *λ* ≥ *λ*_p_ and is characterized by the purely elastic behavior of the spherical shell. In this case, the dimensionless resistance *χ* is defined by the formula:

(9) $$\chi \;=\;\frac{\alpha }{\lambda^{2}}$$

where *α* < 1 is the coefficient of elastic critical resistance due to imperfections. In the case of an ideal shell *α* = 1 and *p*_Rk_ = *p*_Rcr._ For shells operating in this range, it is only possible to exhaust the capacity in the elastic range. In this range *p*_Rk_ = *α* ∙ *p*_Rcr_.

**Figure 21:**
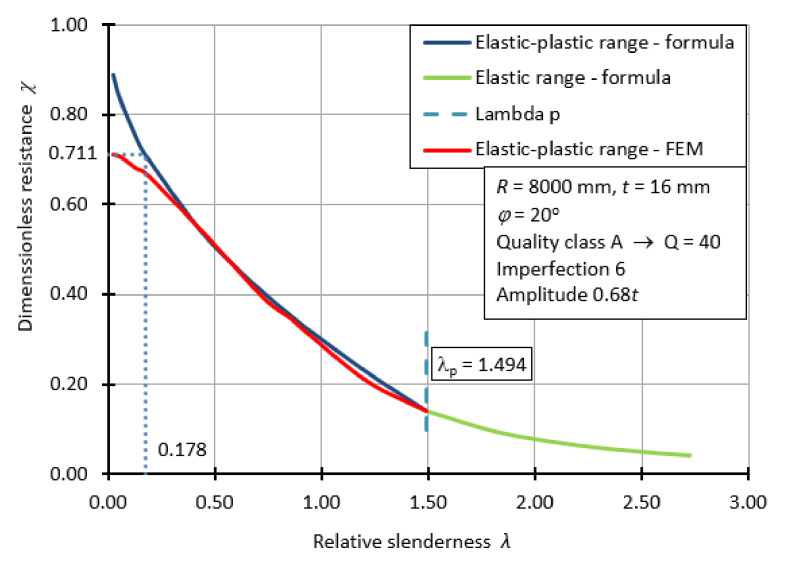
Classical capacity curve for shell with manufactured quality class A.

Figure 21 shows the course of one of the many classical bearing capacity curves for three randomly chosen cases. Such a restriction is again dictated by the very large number of graphs necessary. The example graph corresponds to a spherical shell with fabrication quality class A, angle *φ* = 20°. The shell is subject to imperfection in the form of the first buckling form and its amplitude is 0.68*t*. In creating the classical capacity curve of Figure 21, the buckling parameters *α* and *β* were used and determined from the modified capacity curve (Figure 18), and the direct formula for the exponent *η* value was used in calculating the dimensionless buckling capacity for the elastic-plastic range (formula 8). In addition, the transition point from the elastic-plastic range to the elastic range of the shell operation can be perfectly seen in this diagram. It takes place at the value of *λ*_p_ = 1.494. It is worth noting the fact that on this graph there is no plastic shelf, thus, the value of the buckling parameter *λ*_0_ = 0. In Figure 21, in the interval describing the elastic-plastic range, two curves are also compared with each other. The first one in red is the result of numerical calculations and represents the values of dimensionless buckling capacity obtained from GMNIA and MNA ratio analyses. The second one (blue) shows the dimensionless buckling capacity calculated with Equation (6). In both cases, the resistance curves do not reach 1.0 on the ordinate axis and thus the characteristic plastic shelf does not occur. Moreover, based on FEM calculations, the dimensionless buckling capacity is 0.711, not 1.0 as it would appear from the formula. Therefore, it was necessary to “truncate” the resistance curve determined by the formulae in order to best match the results obtained. In this way, a horizontal section of the bearing capacity curve was created up to a cut-off of 0.178.

**Figure 22:**
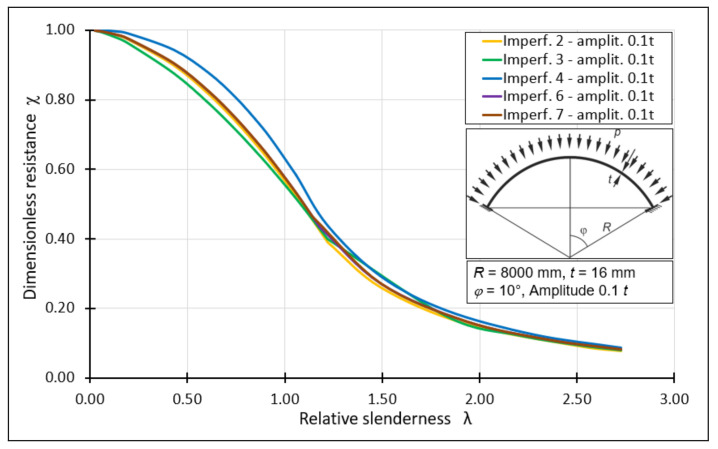

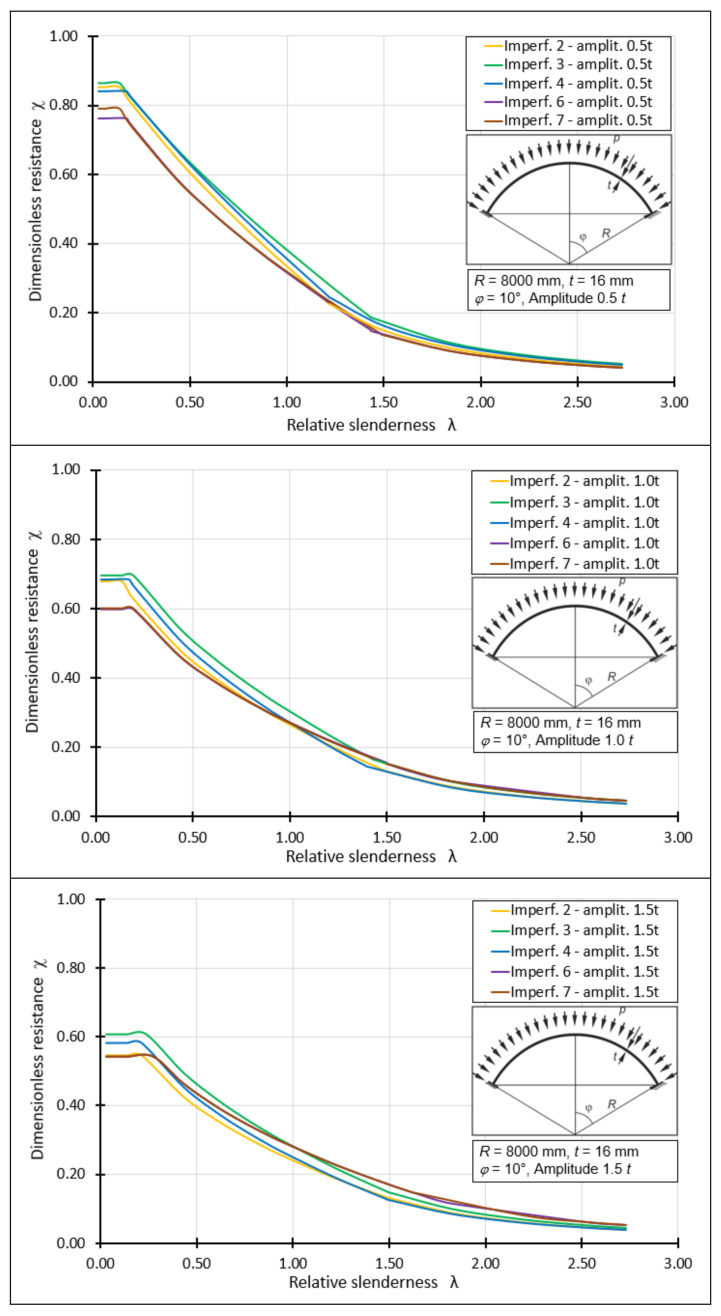

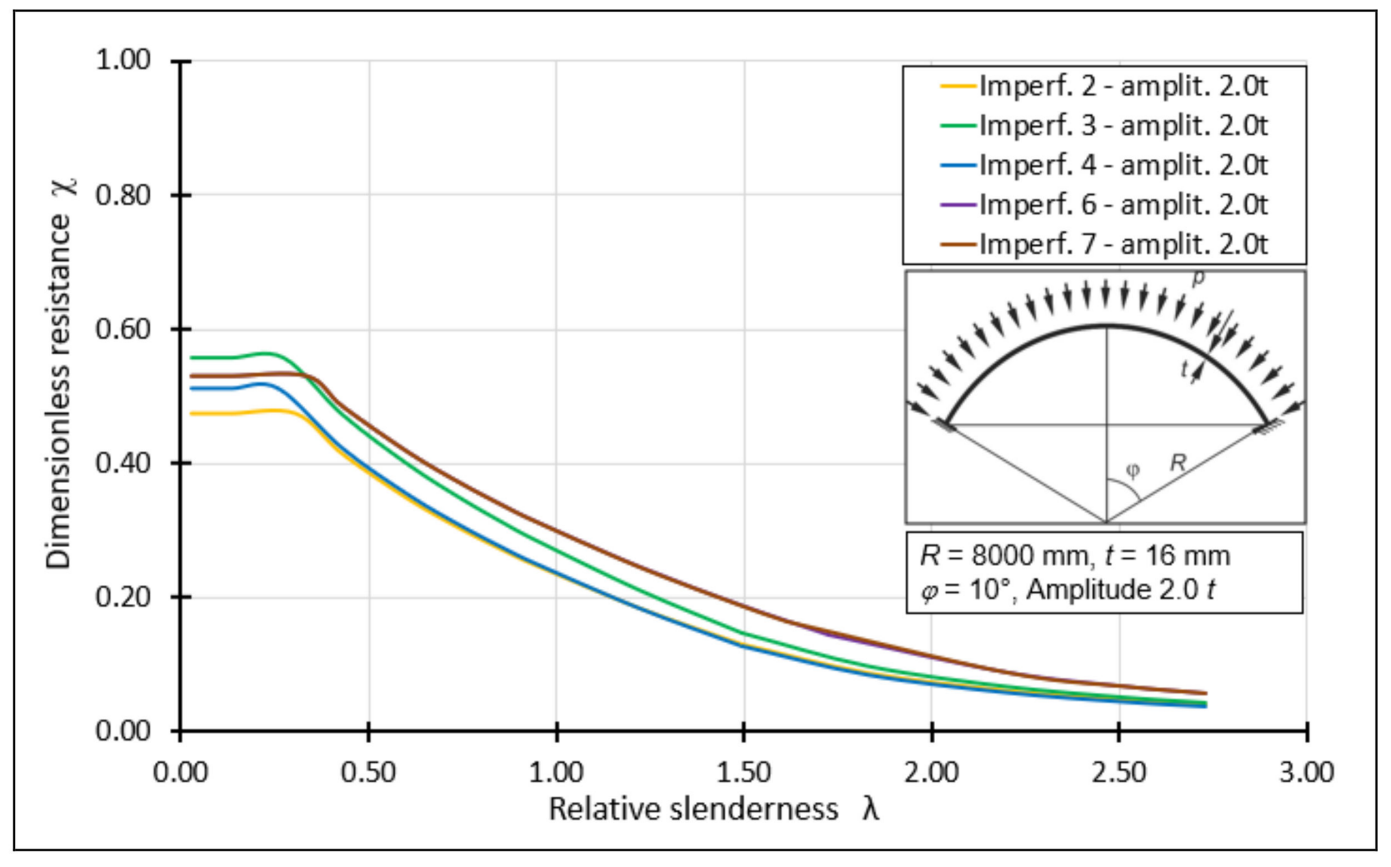
Summary of classical capacity curves for shells with angle *φ* = 10°, different imperfections with different amplitudes.

The procedure presented above was used to create all the individual graphs representing the classical load curves. The next step was to compile the individual curves on one diagram depending on the size of the imperfection amplitude. For this purpose, representative amplitude magnitudes of 0.5*t*, 1.0*t*, 1.5*t*, 2.0*t* were used. Additionally, an amplitude of a very small magnitude, namely 0.1*t*, was analyzed. This was dictated by the fact that the smallest magnitude of the amplitude resulting from the use of manufacturing quality classes was 0.433*t* and is far from an ideal geometry. Figure 22 shows the classical load curves for a spherical shell with half opening angle *φ* = 10°, with various forms of imperfections with declared representative magnitudes of amplitudes. These curves are obtained using classical formulas, describing the dimensionless buckling capacity depending on the shell working range, taking into account the buckling parameters and those obtained from FEM analyses as well as the auxiliary parameter *k*. Only for the occurrence of a very small amplitude of 0.1*t*, a plastic shelf, small in terms of section length, can be observed.

Each successive increase in the imperfection amplitude results in a decrease in the level of the dimensionless buckling capacity. The statement also shows which form of geometrical imperfection has the worst effect on the shell, reducing the dimensionless resistance to the greatest extent. In the case of an amplitude of 0.5*t*, an imperfection 6 in the form of the first buckling form of the system is the most unfavorable. It reduces the dimensionless buckling resistance to the value *χ* = 0.79. Increasing the imperfection amplitude to 1.0*t* also reduces the dimensionless resistance to the value *χ* = 0.60. This value is obtained by two imperfections, the first and the second buckling form. A further increase in the amplitude lowers the dimensionless resistance to a value *χ* = 0.54. In this case, in addition to imperfections 6 and 7, imperfection 2 in the form of a large buckling turns out to be the worst. The occurrence of an amplitude of 2.0*t* results in the reduction in the dimensionless load capacity to the value *χ* = 0.47, and the most unfavorable imperfection is a large surface crush.

The same procedure was followed in creating the sets of classical bearing capacity curves for the other half-shell opening angles.

### 6.4. Modification of the Classic Load-Carrying Curve

Analyzing all the results obtained, in the form of classical buckling load curves for each of the considered spherical shells, one should notice the fact that none of them, burdened with the amplitude from the range of the considered manufacturing quality classes, achieves full plastic load capacity. Such a course of the classical load-carrying capacity curves was also observed in the works of other scientists. This phenomenon is related to the applied method of obtaining different slenderness of spherical shells *λ*. This method consisted of changing the yield stress of *f*_yk_ in order to obtain successive points on the modified load curve. Obtaining a curvy shell (thick) consisted of introducing a very small value of *f*_yk_ while maintaining geometric parameters, obtaining a very slender shell consisted in introducing a very large value of *f*_yk_ while maintaining geometric parameters *R* and *t* and an imperfection amplitude dependent on *t*. The artificially curvy shell had the same imperfection amplitude as the slender shell, and for this reason, the modified load paths did not reach the plateau level. For none of the reference imperfection amplitudes of 0.5*t*, 1.0*t*, 1.5*t*, 2.0*t*, was the characteristic plastic shelf (plateau) obtained, thus the buckling parameter o cannot be determined.

**Figure 23:**
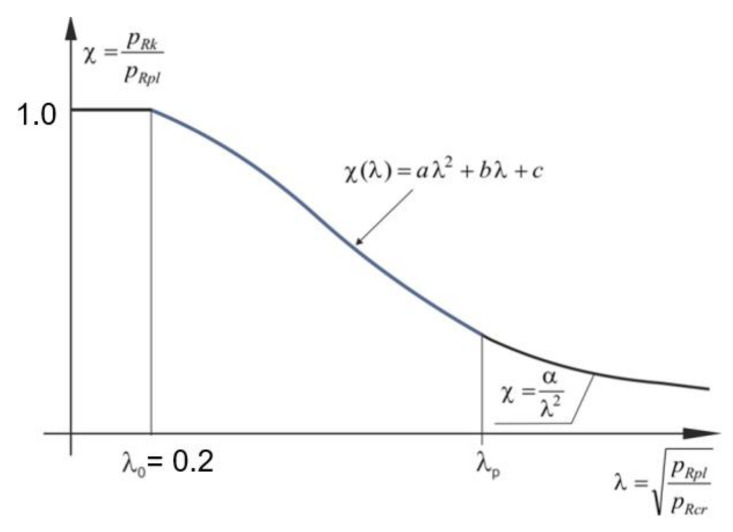
Modification of the classic buckling capacity curve.

Bearing in mind the above considerations and the fact that the classical bearing capacity curves without the characteristic plastic shelf have not been noticed in the literature (Figure 19), in the present work, it was decided to correct the position of the plastic flattening section by modifying the classical bearing capacity curve. The proposal for modification of the classic load-carrying curve is shown in Figure 23.

The plastic shelf is described by the load capacity level deposited on the ordinate axis, which is *χ* = 1.0, and its length by the buckling parameter *λ*_0_ measured on the abscissa axis, which is *λ*_0_ = 0.2. This value was adopted by the authors of the EDR 5th design recommendations and hence this choice. The elastic-plastic range was decided to be described by a second-degree polynomial with continuity at *λ*_0_ and *λ*_p_ points. This change is dictated by a troublesome parameter *η*, which is an exponent of a power when determining the function *χ*(*λ*) in the elastic-plastic range according to PN-EN 1993-1-6. On the other hand, the approach recommended in EDR 5th suggests taking the value of the parameter *η* as *η* = 1.0, which, in the author’s opinion, is an oversimplification. Therefore, it is postulated that on the section <0.2; *λ*_p_ > the curve *χ*(*λ*) has the form:

(10) $$\chi (\lambda )\;=\;a\lambda^{2}\;+\;b\lambda \;+c$$

The coefficients *a*, *b*, and *c* will be found from the conditions:

1. The continuity condition at *λ*_0_ = 0.2

(11) $$\chi \left(\lambda \right)=1\;\;\Rightarrow \;\;\;\;a0.2^{2}\;+\;b0.2\;+c\;=\;1$$

2. The continuity condition at point *λ* = *λ*_p_

(12) $$\chi \left(\lambda \right)=\frac{\alpha }{\lambda^{2}}\;\;\Rightarrow \;\;\;\;\;a\lambda_{p}^{2}\;+\;b\lambda_{p}\;+c\;=\;\frac{\alpha }{\lambda_{p}^{2}}$$

3. Equality condition for the derivatives at point *λ* = *λ*_p_

(13) $$\frac{d\left(\chi \left(\lambda \right)\right)}{d\lambda }=\frac{d}{d\lambda }\left(\frac{\alpha }{\lambda^{2}}\right)\;\;\Rightarrow \;\;\;\;\;2a\lambda_{p}\;+\;b\;=\;\frac{-2\alpha }{\lambda_{p}^{3}}$$

After solving the system of three equations, we obtain:

(14) $$\;a\;=\;\frac{\alpha \left(0.4-3\lambda_{p}\right)+\lambda_{p}^{3}}{\lambda_{p}^{3}\left(0.04\;-\;0.4\lambda_{p}\;+\;\lambda_{p}^{2}\right)\;}\;,$$

(15) $$\;b\;=\;\frac{-2\lambda_{p}^{4}\;+\;\alpha \left(4\lambda_{p}^{2}-0.08\right)}{\lambda_{p}^{3}\left(0.04\;-\;0,4\lambda_{p}\;+\;\lambda_{p}^{2}\right)\;}\;,$$

(16) $$\;c\;=\;\frac{\alpha \left(0.12-0.8\lambda_{p}\right)+\lambda_{p}^{4}\;\;}{\lambda_{p}^{2}\left(0.04\;-\;0.4\lambda_{p}\;+\;\lambda_{p}^{2}\right)\;}\;.$$

The elastic range of the shell operation in the range *λ* > *λ*_p_ remains unchanged and is described in the same way as in the standard in question, namely:

(17) $$\chi (\lambda )=\;\alpha /\lambda^{2}$$

### 6.5. Buckling Parameters

**Figure 24:**
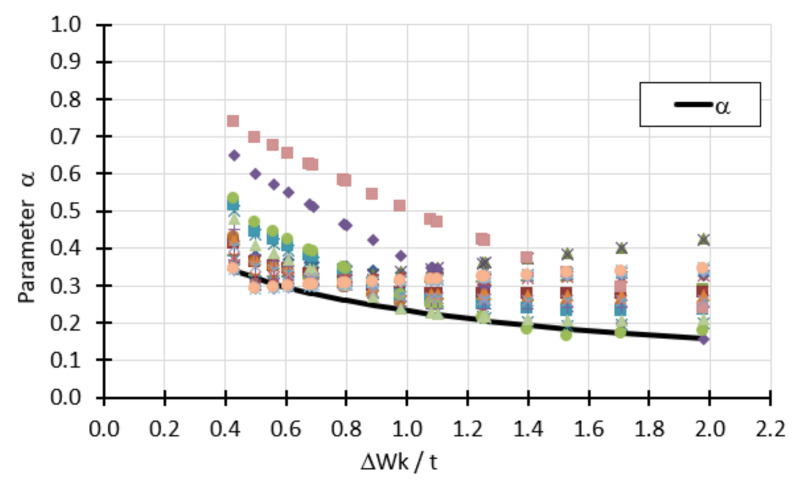
Lower envelope of calculation points describing the parameter.

Proposed changes to the classic capacity curve presented above result in the necessity of specifying only two buckling parameters *α* and *β*. Although the parameter *λ*_0_ determines the extent of the plastic zone, it is not included in any of the proposed formulas. Figure 24 shows calculation points, each of which corresponds to one parameter *α* obtained from the analysis of shells with different angles of opening *φ*, different thickness *t*, different forms of geometric imperfections, different amplitude imperfections, and different manufacturing quality classes.

In order to obtain the greatest reduction in bearing capacity, especially in the elastic range, as well as to ensure safety in all possible cases, the parameter *α* was defined as the lower envelope of the point cloud of Figure 24. This envelope is described by the formula:

(18) $$\alpha \left(\Delta w_{k}/t\right)=\;\frac{0.65}{1\;+\;1.8{\left(\Delta w_{k}/t\right)}^{0.8}}$$

where *Δ*w_k_ is the magnitude of the imperfection amplitude (Equation (3)), *t* is the shell thickness. This formula expresses the dependence on the relative imperfection amplitude. Its form was chosen on the basis of the analogous formula found in EDR 5th, and the coefficients were chosen to describe the lower envelope curve as accurately as possible.

**Figure 25:**
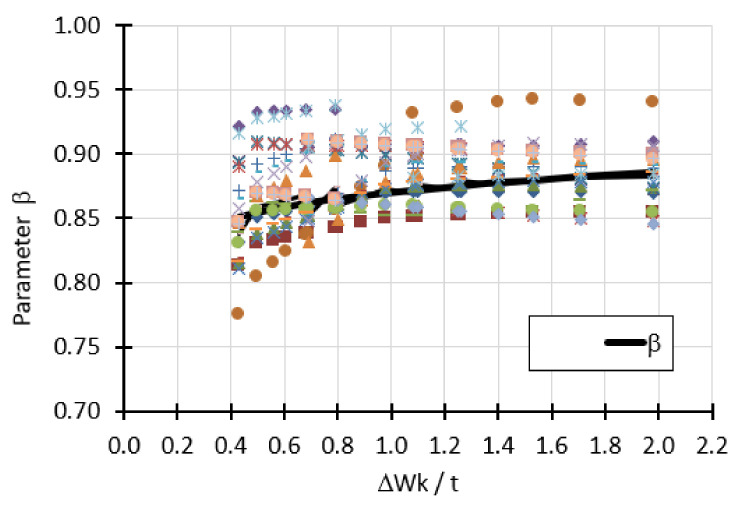
Average values of calculation points describing the parameter.

The second buckling parameter *β* was also determined from multiple design points. They are shown in Figure 25. This time the parameter *β* represents the average values of all the points on the graph as a function of the change in the *Δ*w_k_/*t* ratio.

The curve describing the parameter is written by the formula:

(19) $$\beta \left(\Delta w_{k}/t\right)=\;0.87\;{\left(\frac{\Delta w_{k}}{t}\right)}^{0.026}$$

where *Δ*w_k_ is the magnitude of the imperfection amplitude (Equation (3)), *t* is the shell thickness. This formula expresses dependence *β* on relative imperfection amplitude. Its form was obtained by averaging the values of expressions occurring in the equations describing the trend lines for particular cases depending on the shell thickness *t*, angle *φ*, manufacturing quality parameter *Q*, different forms of geometric imperfections as well as different amplitudes.

It is worth mentioning that both the buckling parameters *α* and *β* obtained depend not only on geometrical parameters of the shell, i.e., radius *R*, thickness *t*, or half opening angle *φ*. They also depend on the manufacturing quality class A, B, C, which influences the value of imperfection amplitude *Δ*w_k_ through the manufacturing quality parameter *Q*.

Having the values of parameters *α* and *β*, it is possible to determine the last buckling parameter which is the relative comparative slenderness *λ*_p_. This is the transition point of the elastic-plastic range to the elastic range of the spherical shell work, defined by the formula:

(20) $$\lambda_{p}=\sqrt{\frac{\alpha }{1-\beta }}$$

## 7. Algorithm for Calculating the Buckling Resistance of Spherical Shells

The above discussion presents the steps necessary to create a procedure for estimating the buckling capacity of spherical shells, which is based on the assumptions of EN 1993-1-6, while maintaining an engineering approach. The procedure is characterized by clarity and its algorithm by a low degree of complexity. In this paper, the author has not changed the main postulates accompanying the dimensioning of spherical shells. The procedure was created on the basis of numerical analyses complying with the norm recommendations and was additionally detailed by issues concerning buckling coefficients. The algorithm of calculation of buckling resistance according to the new procedure is presented below. It can be used to estimate the buckling resistance of spherical shells fixed along the support edge and with elevations for which the half-angle of opening is within the limits of 10° ≤ *φ* ≤ 90°.

Algorithm for calculating buckling resistance of spherical shells:

1. Taking material and geometrical data: *E*, *f*yk, *R, t*
2. Calculation of critical and plastic resistance. Determination of slenderness *λ*:

$$\begin{array}{ccc} {p_{\mathrm{Rcr}}}_{\left(\mathrm{LBA}\right)}=\;1.303E{\left(\frac{t}{R}\right)}^{2} & \\ & \to \;\lambda =\sqrt{\frac{{p_{\mathrm{Rpl}}}_{\left(\mathrm{MNA}\right)}}{{p_{\mathrm{Rcr}}}_{\left(\mathrm{LBA}\right)}}} & \\ {p_{\mathrm{Rpl}}}_{\left(\mathrm{MNA}\right)}=\;1.986f_{yk}\frac{t}{R} & \end{array}$$

3. Selection of shell execution class. Determination of imperfection amplitude:

$$\begin{array}{ccc} \mathrm{Class}\;A\;\to \;Q\;=40 & \\ \mathrm{Class}\;B\;\to \;Q\;=25 & \to \;\Delta w_{k}=\;\frac{1}{Q}\sqrt{Rt} & \\ \mathrm{Class}\;C\;\to \;Q\;=16 & \end{array}$$

4. Determination of buckling parameters *α* and *β*. Determination of slenderness *λ*p:

$$\begin{array}{cc} \alpha \left(\Delta w_{k}/t\right)=\;\frac{0.65}{1\;+\;1.8{\left(\Delta w_{k}/t\right)}^{0.8}} & \\ & \to \;\lambda_{p}=\;\sqrt{\frac{\alpha }{1-\beta }}\\ \beta \left(\Delta w_{k}/t\right)=\;0.87\;{\left(\frac{\Delta w_{k}}{t}\right)}^{0.026} \end{array}$$

5. Determination of working range of spherical shell. Reduction factor *χ*:
  $$\begin{array}{cc} \chi \left(\lambda \right)=\;1, & \mathrm{for}\;\;\lambda <\lambda_{0}\\ \chi \left(\lambda \right)=\;a\lambda^{2}\;+\;b\lambda \;+c, & \;\mathrm{for}\;\lambda_{0}\leq \lambda \leq \;\lambda_{p}\\ \chi \left(\lambda \right)=\;\frac{\alpha }{\lambda^{2}}\;, & \mathrm{for}\;\lambda >\;\lambda_{p} \end{array}$$
  where *λ*_0_ = 0.2

6. Determination of polynomial expressions for elastic-plastic range:

$$\;a\;=\;\frac{\alpha \left(0.4-3\lambda_{p}\right)+\lambda_{p}^{3}}{\lambda_{p}^{3}\left(0.04\;-\;0.4\lambda_{p}\;+\;\lambda_{p}^{2}\right)\;}\;,\;\;b\;=\;\frac{-2\lambda_{p}^{4}\;+\;\alpha \left(4\lambda_{p}^{2}-0.08\right)}{\lambda_{p}^{3}\left(0.04\;-\;0.4\lambda_{p}\;+\;\lambda_{p}^{2}\right)\;}\;,\;\;c\;=\;\frac{\alpha \left(0.12-0.8\lambda_{p}\right)+\lambda_{p}^{4}\;\;}{\lambda_{p}^{2}\left(0.04\;-\;0.4\lambda_{p}\;+\;\lambda_{p}^{2}\right)\;}\;.$$

7. Determination of characteristic buckling capacity of spherical shell:

$$p_{Rk}=\chi \cdot p_{Rpl}$$

## 8. Summary

The stability of spherical shells is an issue that scientists have been working on since the beginning of the 20th century. The first considerations were purely theoretical and led to the determination of the critical load capacity of a full spherical shell. In the second half of the 20th century, experimental studies on real models of spherical shells began. These studies were conducted at several independent research centers on shells made of different types of material with different geometries. The results of the experimental studies allowed the verification of the correctness of the proposed calculation formulas for determining the buckling capacity of spherical shells. The difference between the experimental results and the results obtained from the theoretical approach also became apparent. In order to best reflect the real behavior of spherical shells and thus optimize the process of their design, computer techniques began to be used to an increasing extent. Their constant development led to a situation in which an engineer can model a spherical shell, declare any of its materials, load it, and then perform any numerical analysis determining its critical, plastic, and ultimate load capacity. On the other hand, carrying out a series of numerical analyses of spherical shells with different geometries with additional consideration of the form, location, and amplitude of geometric imperfections allows a more global view of the problem of shell stability, and provides a chance to develop calculation formulas whose results are closer to reality.

This paper presents the process of creating a calculation procedure for estimating the buckling capacity of spherical shells. This procedure is based entirely on the assumptions included in the standard EN-1993-1-6 mentioned—and also becomes a complement of EDR5th by unifying provisions included in them. This procedure is characterized by clarity and its algorithm is characterized by a low degree of complexity. While developing the procedure, no attempt was made to change the main postulates accompanying the dimensioning of spherical shells. The result is an engineering approach to a very complex problem. In spite of the simple calculation algorithm for estimating the buckling capacity of spherical shells, the results obtained reflect the behavior of real spherical shells, regardless of their geometry and the material used to manufacture them, extremely accurately. This is confirmed by comparing the newly developed procedure with experimental studies, in which the experimental results are almost identical to those obtained using the calculation formulas. This comparison is all the more valuable because the experimental studies were conducted on samples made of different materials, and the ratio of radius *R* to shell thickness *t* almost completely exhausted the range that could be used in practice. In the following articles, the developed procedure for estimating the buckling capacity of spherical shells will be compared with the current provisions of EN-1993-1-6 and the recommendations of EDR 5th as well will be compared with the experimental studies performed so far. 

## Figures and Tables

**Table 1:** Critical pressure *p*_Rcr_ [MPa].

			Half-Angle of Opening *φ*					
*R* [mm]	*t* [mm]	*R/t*	*φ* = 10°	*φ* = 20°	*φ* = 30°	*φ* = 45°	*φ* = 60°	*φ* = 90°
8000	8.000	1000	0.2653	0.2658	0.2652	0.2611	0.2588	0.2564
8000	10.667	750	0.4742	0.4722	0.4731	0.4674	0.4633	0.4597
8000	13.333	600	0.7472	0.7362	0.7381	0.7369	0.7288	0.7236
8000	16.000	500	1.0849	1.0605	1.0631	1.0603	1.0513	1.0441
8000	20.000	400	1.7038	1.6546	1.6602	1.6628	1.6575	1.6422
8000	26.667	300	3.1178	2.9486	2.9446	2.9527	2.9483	2.9461

**Table 2:** Reference plastic resistances *p*_Rpl_ [MPa].

			Half-Angle of Opening *φ*					
*R* [mm]	*t* [mm]	*R/t*	*φ* = 10°	*φ* = 20°	*φ* = 30°	*φ* = 45°	*φ* = 60°	*φ* = 90°
8000	8.000	1000	0.4714	0.4660	0.4616	0.4571	0.4548	0.4472
8000	10.667	750	0.6304	0.6233	0.6176	0.6119	0.6071	0.6019
8000	13.333	600	0.7905	0.7808	0.7741	0.7672	0.7626	0.7574
8000	16.000	500	0.9516	0.9381	0.9310	0.9228	0.9175	0.9127
8000	20.000	400	1.1953	1.1743	1.1666	1.1566	1.1504	1.1455
8000	26.667	300	1.6084	1.5688	1.5604	1.5476	1.5396	1.5336

**Table 3:** Values of the manufacturing quality parameter.

Manufacturing Quality Class	Description	Generation Quality Parameter Q
Class A	Excellent	40
Class B	High	25
Class C	Normal	16

## Data Availability

Not applicable.
